# Treatment of Facial Asymmetry Caused by Parry-Romberg Syndrome Using Fat Transfer

**DOI:** 10.7759/cureus.80977

**Published:** 2025-03-21

**Authors:** Evelyn C Echevarria Cruz, Brooke E Heyer, Jamie M Moenster

**Affiliations:** 1 Medicine, Dr. Kiran C. Patel College of Osteopathic Medicine, Nova Southeastern University, Fort Lauderdale, USA; 2 Plastic Surgery, Dermatology and Plastic Surgery of Arizona, Tucson, USA

**Keywords:** fat grafting, fat transfer, parry-romberg syndrome, plastic surgery, progressive hemifacial atrophy

## Abstract

Parry-Romberg syndrome (PRS) is a rare craniofacial disorder marked by progressive unilateral facial atrophy. This case report presents a unique instance of PRS presenting in a 52-year-old female who was diagnosed at age 47 as the syndrome’s usual onset is during the first two decades of life. The patient exhibited severe soft-tissue atrophy on the right side of the face, classified as type 3 PRS, along with associated symptoms such as headaches and a history of depression. The primary aesthetic intervention involved fat transfer (FT) to restore facial symmetry and contour. Postoperative outcomes improved facial aesthetics, though some volume loss was noted at the three-month follow-up. This case highlights the potential of FT as an effective and minimally invasive treatment for severe PRS. It emphasizes the importance of personalized treatment strategies and the need for further research into the long-term management of PRS, particularly in late-diagnosed cases. Further studies are needed to optimize fat grafting techniques and assess long-term outcomes in PRS management.

## Introduction

Parry-Romberg syndrome (PRS), also known as progressive hemifacial atrophy, is a rare, acquired craniofacial disorder characterized by persistent unilateral facial atrophy. PRS typically manifests within the first two decades of life, with a slow progression lasting 2 to 10 years before stabilization [1]. The condition primarily affects the skin, subcutaneous tissue, and fat, but in advanced stages, it can extend to involve muscle and bone [2]. It is estimated to affect approximately one in 700,000 individuals and occurs more frequently in females, though both genders can be affected [3,4]. As the syndrome progresses, extracutaneous manifestations may occur, including neurologic, ophthalmic, cardiac, dental, and maxillofacial complications. Common clinical features include seizures, headaches, trigeminal neuralgia, enophthalmos, delayed tooth eruption, and temporomandibular joint dysfunction [1]. We present a rare case of PRS with a late-onset presentation and severe facial atrophy, successfully treated with fat transfer (FT). While FT is typically used for mild to moderate cases, its effectiveness in severe PRS remains underreported. This case expands the current understanding of PRS management by demonstrating the potential of FT as a minimally invasive treatment alternative, highlighting its role in improving facial volume restoration even in late presentations.

## Case presentation

A 52-year-old Caucasian female presented to the plastic surgery office for consultation regarding facial rejuvenation due to the loss of facial volume in the right lower face, attributed to PRS, and general dissatisfaction with her facial appearance. Her past medical history includes depression, breast cancer treated with radiation and a double mastectomy with reconstruction, and diagnosis of PRS in 2017, which has been associated with headaches and migraines. Additionally, her family history reveals several relatives with autoimmune disorders.

**Figure 1:**
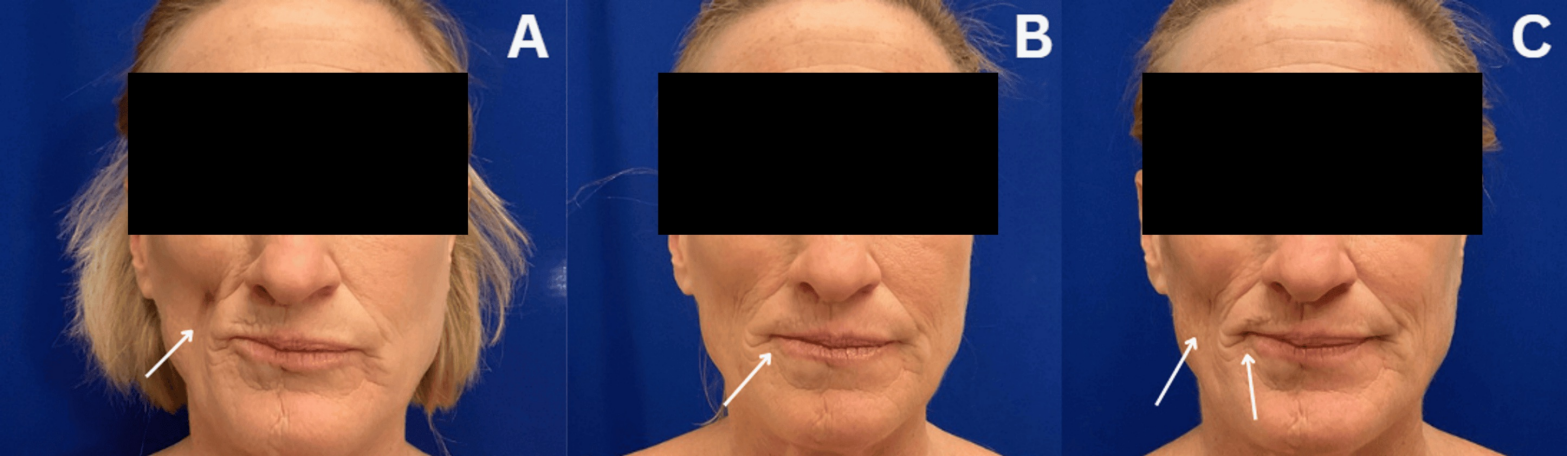
Clinical presentation 1A. Pre-operative photo with an arrow indicating soft tissue atrophy on the right side of the mid-lower face, along with moderate descent of the right cheek, malar fat pad, and volume loss in the pre-jowl sulcus; 1B. One-month postoperative photo with an arrow demonstrating appropriate healing and some volume loss to the lips; 1C. Three-month postoperative photo with an arrow on the left side of the image indicating mild volume loss in the right cheek and a centrally positioned arrow demonstrating mild volume loss to the lips.

During the physical examination, findings indicated soft tissue atrophy on the right side of the mid-lower face and neck, moderate descent of the right cheek, malar fat pad, and volume loss in the pre-jowl sulcus, all secondary to PRS (see Figure 1A). Due to the extent of involvement of soft tissue, cartilage, and bony atrophy, this patient was determined to have severe or type 3 PRS.

After a comprehensive discussion and a careful review of her medical history, surgical intervention was determined to be the most beneficial approach. FT was performed, utilizing 39 cc of fat harvested from the abdomen to enhance volume in the right side of the face, specifically to the lips and cheek.

To obtain the necessary graft material, a fat harvesting procedure was performed using a tumescent solution consisting of 28 cc of 1% lidocaine with epinephrine, injected into the abdomen to minimize discomfort and bleeding. After allowing time for vasoconstriction, 900 cc of fat was aspirated for future transfer. The harvested fat was processed using the "Red Head" FT system, which employs decantation and filtration with saline to optimize graft viability. Aspiration was performed with 3 mm and 4 mm cannulas, while FT was conducted using a 17-gauge Tulip single-opening cannula. Moreover, preoperative IV antibiotic prophylaxis was administered to reduce the risk of infection. Notably, more fat was harvested than required for facial transfer alone, as an additional but separate surgical procedure performed during the operation required additional fat grafting.

One week following the procedure, her incisions had healed well without signs of infection, and the facial contour appeared very satisfactory. By one month post intervention, the patient was healing appropriately; however, some volume loss from the FT in the lips was noted as expected (see Figure 1B).

At the three-month follow-up, mild volume loss persisted (see Figure 1C), prompting discussions regarding follow-up procedures, including the potential use of additional FT or considering adding dermal fillers with focus to the lateral commissure, upper and lower lips, and mid-cheek. An agreement was reached to monitor her healing and reassess in three to six months.

## Discussion

We report a case of PRS in a 52-year-old female treated with FT to atrophied areas to restore symmetry and contour. PRS is a rare condition affecting an estimated one in 700,000 individuals, but the actual incidence is unknown [3]. It is more commonly seen in women [4]. The pathophysiology of PRS is poorly understood and has an unclear cause, but evidence suggests a multifactorial origin. It is often connected to autoimmunity due to its association with autoimmune diseases such as scleroderma and the presence of autoantibodies [1]. There may also be a genetic component, with some cases indicating autosomal dominant inheritance with incomplete penetrance. Potential triggers for PRS include trauma, infections, and neurovascular dysfunction. Histopathology indicates skin atrophy, fibrosis, lymphocytic infiltration, and vascular degeneration, suggesting an inflammatory or immune-mediated process [2].

PRS is categorized as mild, moderate, or severe based on the number of trigeminal nerve branches involved. Mild cases affect only one branch, moderate cases involve two branches, and severe cases impact all three branches of the trigeminal nerve with atrophy progression to the entire face [1,5]. The condition can also be classified into four types. Type 1 is the mildest, with minimal thinning of facial soft tissue. Type 2 involves more moderate facial soft tissue thinning but no involvement of cartilage or bone. Type 3 has even thinner soft tissue than type 2, with early thinning of the cartilage and bone, and type 4 is the most severe, with significant soft tissue atrophy, very thin cartilage and bone, and functional deficits, especially with the lips and nose [6].

Diagnosing patients with PRS presents a significant challenge due to the rare presentation of disease, similarity to and overlapping with other pathologies, and the variability in patient presentation ranging from musculoskeletal, ophthalmological, and neurologic symptoms. The diagnosis of PRS is made through clinical evaluation and physical examination and is supported by imaging with CT scan or MRI. Additionally, histopathological examination of the skin typically reveals atrophy of the epidermis, dermis, subcutaneous tissue, and vessels, along with skin fibrosis, inflammation, and edema [2]. The hallmark features of PRS are soft tissue atrophy with variable hyperpigmentation of the skin in addition to various extracutaneous findings such as migraine headaches, trigeminal neuralgia, enophthalmos, and epilepsy [1,7]. In addition to the physical findings, the patient's social and psychological well-being can be impacted due to this disfiguring disease. If necessary, medical management and surgical intervention are recommended to be started early after diagnosis as prompt treatment can slow disease progression, maximize quality of life, and minimize psychological stress in patients [8].

These findings were consistent with our patient's presentation of severe, type 3 PRS associated with headaches and migraines and a history of depression. Notably, PRS is typically diagnosed in the first two decades of life. However, in the case of our patient, she was diagnosed in the fourth decade of life and being followed by a rheumatologist for her condition.

Treatment for PRS typically begins with immunosuppressive drugs to help stabilize the disease and slow its progression. Aesthetic treatment is typically recommended to start one to two years after disease stabilization [1]. Patients who are found to have an autoimmune etiology or those with co-existing autoimmune conditions are commonly started on a combination of immunosuppressive agents such as corticosteroids and methotrexate [8]. One case reported treatment of PRS with secukinumab, an interleukin-17 (IL-17) inhibitor, with apparent successful halting of progression, which clues into the autoimmune pathology of the disease [9].

Aesthetic interventions for PRS patients may include dermal and autologous fat grafts, soft tissue and muscle flap grafts such as free differential-thickness adipose-fascial anterolateral thigh flaps and galeal flap grafts, also microsurgical flap grafts incorporating dermis, fat, and muscle tissue, free silicone injections, and bone augmentation [6,10]. Another possible treatment option that has demonstrated various degrees of longevity from 6 to 24 months in PRS and is a simple way to correct facial asymmetry is dermal fillers such as poly-L-lactic acid and hyaluronic acid fillers [11,12]. However, fat grafts are commonly used and favored in patients due to their minimal invasiveness, the procedure's safety profile, the ability to be performed in office or in the operating room, and satisfactory aesthetic outcome. They can restore facial symmetry and contour, and have a positive impact on patients' psycho-social well-being [8]. Additionally, evidence suggests PRS shares similarities with scleroderma and is often found in patients diagnosed with scleroderma [13]. Fat grafting in patients with scleroderma has proven beneficial for soft tissue atrophy [14].

Regarding whether any grade of PRS can be treated with FT, while FT can be effective in all grades of PRS, its efficacy may be limited in severe cases (type 4), where there is significant involvement of the bone and cartilage creating functional deficits of face or jaw. In such cases, adjunctive procedures like flap surgery might be necessary for better structural support. However, our patient opted against flap surgery, as her concerns were purely aesthetic, with no functional deficits at the time of presentation.

One of the limitations of fat grafting is the gradual loss of volume over time after injection, which was observed in our patient's case. One study by the American Society of Plastic and Reconstructive Surgery concluded that around 30% of the adipose tissue remained viable one year after transfer [15]. In clinical studies combining autologous fat with stem cells, the graft gained 55% survival at one-year post procedure, and other studies demonstrated up to 83% fat survival but included multiple transfer sessions [16,17]. Future research focusing on high-resolution CT scan imaging and 3D printing as an added tool for procedural and surgical preparation could significantly benefit patients with PRS by mapping the area needing FT and estimating how much fat is necessary [18]. This could aid in further fat survival and faster yet more precise procedures, ultimately leading to better aesthetic outcomes. Another limitation of our case is the short-term follow-up, as our patient was evaluated only at one month and three months post treatment. Longer follow-up is necessary to assess the durability and final aesthetic outcome of FT in severe PRS cases.

For patients experiencing volume loss over time following FT, repeat FT may be necessary, as observed in our case. Multiple sessions may be required to achieve and maintain a satisfactory aesthetic outcome. Long-term monitoring and consideration of adjunctive procedures after the initial fat grafting can help optimize results.

A potential treatment algorithm for PRS could be based on disease severity. Mild cases (types 1 and 2) may be effectively managed with FT, dermal fillers, or other minimally invasive options. As severity increases (types 3 and 4), FT remains a primary approach, with dermal fillers as possible adjuncts. However, flap surgery should be considered for patients with significant volume loss, especially in cases of severe cartilage or bone destruction where FT alone may be insufficient for structural support.

It is important to note that BMI changes before and after FT may contribute to longevity of autologous fat grafting. Our patient weighed 84.0 kg at the pre-operative visit, and by the three-month postoperative visit, she weighed 80.2 kg. A change of 3.8 kg, in which she decreased her BMI from 27.3 kg/m^2^ to 26.1 kg/m^2^, could have contributed to her loss of volume post procedure.

## Conclusions

PRS remains an understudied disease with unknowns about its etiology and disease course. In this report, we presented the case of a 52-year-old female with PRS causing loss of facial volume to her right lower face. The patient was successfully treated with FT, a minimally invasive procedure that provided noticeable improvement in facial symmetry. While the outcome was positive, some volume loss occurred over time, highlighting the need for continued monitoring and potential follow-up interventions. Currently, much of the reported clinical data about PRS is found through case reports, leading to difficulty in making conclusions about the disease. Future research should include following patients for more extended periods from the diagnosis to obtain more data to better understand disease onset and progression. Further research is needed to explore the disease's genetic factors and potential associations with other conditions. Advances in imaging technology, including high-resolution CT scan and 3D facial implant, may offer opportunities to further enhance outcomes for PRS patients by potentially offering personalized treatment strategies.
